# A multiscale compartment-based model of stochastic gene regulatory networks using hitting-time analysis

**DOI:** 10.1063/5.0010764

**Published:** 2021-05-11

**Authors:** Adrien Coulier, Stefan Hellander, Andreas Hellander

**Affiliations:** Department of Information Technology, Uppsala University, Box 337, SE-755 01 Uppsala, Sweden

## Abstract

Spatial stochastic models of single cell kinetics are capable of capturing both fluctuations in molecular numbers and the spatial dependencies of the key steps of intracellular regulatory networks. The spatial stochastic model can be simulated both on a detailed microscopic level using particle tracking and on a mesoscopic level using the reaction–diffusion master equation. However, despite substantial progress on simulation efficiency for spatial models in the last years, the computational cost quickly becomes prohibitively expensive for tasks that require repeated simulation of thousands or millions of realizations of the model. This limits the use of spatial models in applications such as multicellular simulations, likelihood-free parameter inference, and robustness analysis. Further approximation of the spatial dynamics is needed to accelerate such computational engineering tasks. We here propose a multiscale model where a compartment-based model approximates a detailed spatial stochastic model. The compartment model is constructed via a first-exit time analysis on the spatial model, thus capturing critical spatial aspects of the fine-grained simulations, at a cost close to the simple well-mixed model. We apply the multiscale model to a canonical model of negative-feedback gene regulation, assess its accuracy over a range of parameters, and demonstrate that the approximation can yield substantial speedups for likelihood-free parameter inference.

## I. INTRODUCTION

Including noise in biological models has proven essential in order to better understand the cost and constraints of gene regulation.^1^ Such noise can come from various sources. For example, extrinsic noise arises from small variations in the experimental setup, such as temperature or pH, affecting the biochemical parameters of the cell. Intrinsic noise on the other hand is a result of low concentrations of molecules and the discrete nature of chemical reactions, that is, molecules randomly diffusing and reacting upon collision. The latter has received large interest in molecular systems’ biology, and for this reason, discrete stochastic models of chemical kinetics are often used to model gene regulatory networks in living cells.^2–8^

A wide collection of numerical methods has been developed for discrete stochastic chemical kinetics, spanning various levels of sophistication, computational cost, and accuracy,^9^ and in many cases showing qualitative or even quantitative agreement with experimental data. The Stochastic Simulation Algorithm (SSA), also known as Gillespie’s Algorithm,^10^ is the most widely used method to simulate models formulated as continuous-time discrete-space Markov (CTMC) processes, and many refinements and approximations of the SSA have been proposed.^11^ The random time change representation^12^ offers a complementary view and has also been the basis for extensions and improved algorithms, notably to accommodate time-dependent propensities and delays.^13^ Often, it is assumed that the system is well-mixed. In this context, molecular diffusion is assumed to be fast relative to biochemical reactions so that the system is reaction-limited. In the CTMC framework, the biochemical system is modeled with the chemical master equation (CME),

$$\begin{array}{ll} \frac{\partial P}{\partial t}\left(x,t|x_{0},t_{0}\right) & =\overset{M}{\underset{j=1}{\sum }}a_{j}\left(x-\nu_{j}\right)P\left(x-\nu_{j},t|x_{0},t_{0}\right)\\ & \;-a_{j}\left(x\right)P\left(x,t|x_{0},t_{0}\right), \end{array}$$ (1)

where ***x*** is the state vector. Each row in ***x*** represents the copy number of a given species. *P*(***x***, *t*|***x***_0_, *t*_0_) is the probability density of that state vector, where ***x***_0_ is the initial state and *t*_0_ is the starting time. *a*_*j*_ and ***ν***_*j*_ are the reaction rate and stoichiometric coefficients of reaction *j*, respectively. Unfortunately, it is, in general, computationally intractable to solve the CME due to the exponential growth of the number of states with increasing dimension (the curse of dimensionality). Monte Carlo methods using the SSA to generate realizations of the CTMC are often the only viable alternative to produce an approximate solution to this equation.

In some cases, the well-mixed assumption is not valid. If diffusion is slow relative to reactions, spatial correlations influence the kinetics and can lead to, e.g., cluster formation or spatial bi-stability.^14,15^ In other cases, the system may include spatial features, such as membranes or macromolecular crowding,^16,17^ that must be included in the model for accurate results. For such systems, spatial stochastic models are needed. Both microscopic particle-tracking methods and mesoscopic discrete stochastic methods are widely used.

eGFRD (enhanced Green’s Function Reaction Dynamics) is an efficient exact method for simulating particles in continuous space and time following Smoluchowski diffusion-limited reaction kinetics.^18^ For efficiency, protective domains containing exactly one or two molecules are constructed. The random exit time from these domains is then sampled, and an event queue is built from exit, diffusive, and reaction events between molecules in the same protective domain. The method relies on analytical solutions of the Smoluchowski equation (which involves Green’s functions) to guarantee competitive performance; however, these are not always available, especially for complex geometries.

Another class of methods, used, for instance, in Mcell^19^ and Smoldyn,^20^ tracks the position of all the molecules of interest in the system in mesh-free, continuous space, but discretizes time. Here instead, the random new position of each particle is sampled at *t* + Δ*t* according to Smoluchowski’s dynamics. These methods can be more efficient than eGFRD and can handle complex geometries, at the cost of a discretization error.

On the mesoscopic level, the reaction–diffusion master equation (RDME) framework^21^ relies on space being discretized into voxels, where each voxel is considered to be well-mixed. Diffusion is modeled by allowing each molecule to move from voxel to voxel with a jump rate depending on the diffusion constant and the mesh.^22^ The system can then be simulated using versions of the SSA optimized for the reaction–diffusion case such as the Next Subvolume Method (NSM),^23^ where events are either a chemical reaction localized to a specific voxel or a diffusive jump between a pair of adjacent voxels. Originally demonstrated on Cartesian meshes, the RDME has been extended to unstructured meshes,^22^ allowing for simulations in complex geometries. Depending on the needed mesh resolution, the RDME model can be much faster than microscopic models, although there are additional numerical considerations that can make the method hard to use reliably for non-experts, in particular, related to the limiting behavior as the mesh size goes to zero.^23–25^ In addition, for spatial simulations, approximate and hybrid methods have been developed, e.g., by assuming that some species copy numbers are not too small so that tau-leaping can be used.^26,27^

RDME simulations are, in general, efficient if the mesh can be chosen appropriately; however, to correctly resolve boundaries, the mesh cannot be too coarse near those boundaries. A varying mesh size in the domain can be used, but the mesh size cannot be varied too rapidly locally since this can lead to poor mesh quality that can negatively influence accuracy.^28^ In addition, the technical debt and software footprint of a RDME system are relatively high, software needed to handle mesh generation, assembly of diffusion jump matrices, etc.^29^ This is not a major issue when the objective is to carefully study the detailed spatial aspects of cellular control systems in single cells, such as in Refs. 4, 14, 30, and 31. However, there are important scenarios where also RDME simulation becomes too expensive and where simpler, more specialized multiscale approximation that captures key aspects of the spatial dynamics without the full complexity of needing a mesh could be very valuable. Tasks such as likelihood-free parameter inference or sensitivity analysis are good examples, where a large number of repeated simulations are needed. Another important scenario that calls for cheaper approximations is when embedding a spatial stochastic gene regulation model in a multicellular simulation. Center-based models (CBMs) are the most commonly used frameworks in that domain, with hundreds of thousands of cells approximated geometrically as spheres interact mechanically. As an example, we recently developed a multicellular model of cancer tumor growth where Smoldyn was used to model intracellular spatial dynamics in order to study the effects of chemical kinetics on tumor growth rate.^32^ Due to the large computational cost, simulations were limited to a small number of cells (10–100).

The goal in this paper is to develop and demonstrate a systematic way to construct multiscale models of stochastic gene regulation that capture key aspects of the spatial dynamics without a fully resolved particle-based or mesh-based spatial stochastic simulation. Our driving requirements are that the approximation should not rely on mesh generation (to facilitate future embedding in, e.g., center-based models) and that the computational cost should be very close to a corresponding simplistic well-mixed model (WMM). Furthermore, we want to be able to easily parametrize the approximation *a priori* without any significant pre-processing for the important special case where the geometry is made up of two concentric spheres modeling the cytoplasm and the nucleus. This geometry is important since it is widely used to model signal transduction in yeast and mammalian cells; see, e.g., Refs. 30 and 31. It is also the geometry used in multicellular center-based models. We approach this by formulating a coarse-grained, compartment-based well-mixed model and by obtaining its parameters using hitting-time analysis on the Smoluchowski diffusion-limited model. Using this strategy, we are able to speedup simulations compared to a detailed spatial simulation, while maintaining key features of the higher modeling fidelity compared to a naive well-mixed or compartment-based model. We derive easy-to-evaluate reaction rates for the compartment-based model for the important special case of two concentric spheres modeling the cytoplasm and the nucleus and explain how the parameters can be computed using finite element methods for general geometries. The key benefit of our proposed approximation is that we obtain a very cheap surrogate model that can be used to improve computational efficiency in tasks such as likelihood-free parameter inference. We note that the proposed model is not intended as a general replacement of the Smoluchowski model or of the RDME framework when high spatial resolution is needed, but rather as a cheap alternative to a well-mixed model that adds some spatial detail with very limited increase in cost or code complexity.

Negative feedback arising from self-repression is an omnipresent motif in models of gene expression. It is often used as a component in larger pathways and has been studied extensively in previous modeling work, including in the spatial stochastic setting. For example, Sturrock *et al.*^50^ used the motif to develop a spatial stochastic model of the Hes1 gene regulatory network in embryonic stem cells, showing that unlike well-mixed models, it is capable of reproducing the behavior of wild-type embryonic stem cells^34^ and gives insights into the role of noise in cell differentiation. A known property of the stochastic feedback loop is the bursty gene expression and the possibility of oscillations in protein and mRNA copy numbers.^35^ We use the negative-feedback motif as a case-study throughout the paper.

The remainder of this paper is organized as follows. In Sec. II, we introduce the model of negative feedback and briefly review well-mixed and spatial stochastic chemical kinetics models commonly in use in systems biology and compare these two approaches quantitatively. In Sec. III, we introduce the proposed multiscale compartment model and explain how to parametrize it based on the microscopic model. Section IV evaluates the approximation in terms of accuracy and speedup over a spatial particle-based simulation using Smoldyn, discussing the domain of applicability of the proposed model. We also demonstrate the possible computational gain in the application of parameter inference. Finally, in Sec. V, we conclude the paper with a discussion of possible applications in more complex modeling tasks and possible extensions of the method.

## II. BACKGROUND

In this section, we first introduce the negative-feedback model. We briefly then review the well-mixed and spatial stochastic model formalism and give examples of how trajectories and distributions generated from the models compare in different areas in parameter space. Finally, we compare simulations of a spatial stochastic representation and a discrete well-mixed representation quantitatively over a wider range of parameters.

### A. Model of negative-feedback gene regulation

**FIG. 1.**
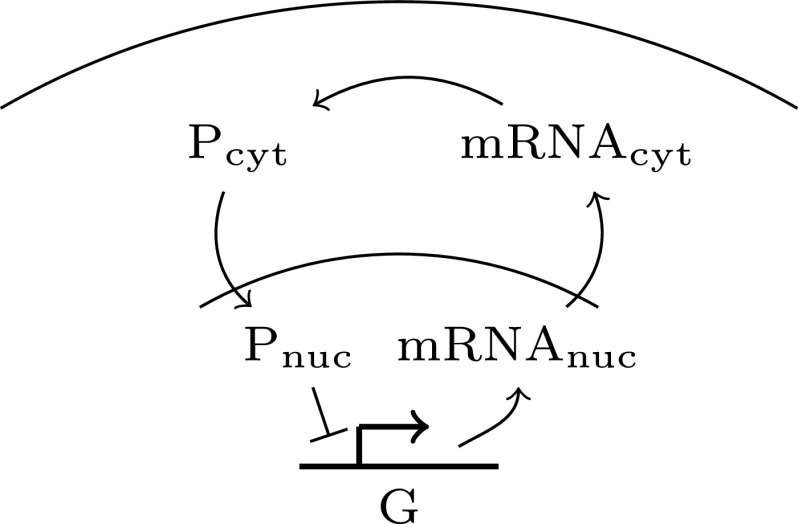
Sketch of the negative-feedback loop. The delayed response induced by compartmentalization and spatial localization of chemical species in the reaction network results in a bursty or oscillatory expression of the gene. It is critical to capture diffusive transport of molecules between compartments and the spatial stochastic nature of the chemical kinetics in order to capture realistic system behavior.^33^

The stochastic spatial model we consider was first proposed in Ref. 33 to study the impact of noise in embryonic stem cell differentiation using the RDME. As illustrated in Fig. 1, a gene placed in the nucleus at the center of the cell transcribes mRNA. The mRNA molecules then diffuse out of the nucleus and into the cytoplasm. There, they get translated into proteins. These proteins then diffuse back into the nucleus and bind to the gene, suppressing their own expression. This creates a delayed response between mRNA transcription and gene repression, giving rise to stochastic oscillations in the latter. This canonical model of negative-feedback gene regulation is a suitable test problem for method development, since the motif is both of direct interest to modelers^33,36,37^ and it frequently appears as a module in larger, more complex networks, such as models of Delta-Notch signaling.^38,39^ In particular, the negative-feedback model has recently highlighted the need to capture both stochastic and spatial effects for qualitative *and* quantitative understanding of Hes1 gene regulation.^31,33^

The chemical reactions are described in Eqs. (2)–(6), where $G_{\emptyset }$ and *G*_*P*_ are the gene promoter sites in unbound and bound states, respectively. Reactions (2) and (3) are restricted to the nucleus, while reaction (4) is restricted to the cytoplasm. Reactions (5) and (6) can fire everywhere in the cell. In our model, the nuclear membrane acts as a barrier, only allowing proteins to enter the nucleus from the cytoplasm and mRNA to exit the nucleus into the cytoplasm. This is a common way to model the membrane and is used in Ref. 33. More complex treatment of the membrane transport is of course possible, for example, by explicitly modeling nuclear pores, but in a RDME model in Ref. 31, such an addition showed no major impact on the overall system dynamics. The baseline parameters of our model are summed up in Table I and are taken from Sturrock *et al.*,^31^

$$G_{\emptyset }+P\;\underset{k_{d}}{\overset{k_{a}}{⇌}}G_{P},$$ (2)

$$G_{\emptyset }\overset{\mu }{\to }G_{\emptyset }+mRNA,$$ (3)

$$mRNA\overset{\kappa }{\to }mRNA+P,$$ (4)

$$mRNA\overset{\gamma }{\to }\emptyset ,$$ (5)

$$P\overset{\gamma }{\to }\emptyset .$$ (6)

**TABLE I.** Base parameters as presented in Sturrock *et al.*^33^ Parameters in bold are varied across several orders of magnitude to compare our models in different configurations (Sec. IV). Throughout this study, *μ*, *κ*, and *γ* are varied simultaneously by multiplying them by a common variable, noted *χ*.

Parameter	Description	Localization	Base value
*R*	Cell radius		6.0 *µ*m
*r*_*n*_	Nucleus radius		2.5 *µ*m
*σ*	Molecular radius		0.01 *µ*m
***D***	Diffusion constant		0.6 *µ*m^2^ min^−1^
*k*_*a*_	Binding rate	Nucleus	1.00 × 10^9^ m^−1^ min^−1^
*k*_*d*_	Unbinding rate	Nucleus	0.1 min^−1^
***μ***	Transcription rate	Nucleus	3.0 min^−1^
***κ***	Translation rate	Cytoplasm	1.0 min^−1^
***γ***	Degradation rate	Entire cell	0.04 min^−1^

This simple regulatory network is found in many biological systems. It is inherently spatial due to the localization of reactions. However, the degree of spatial effects depends on the parameters of the model, in particular, on the degree of diffusion control of the reactions. Simply put, the faster molecular diffusion is relative to bimolecular association, the more well-mixed the system will be. Since the spatial stochastic simulations are much more computationally expensive than their well-mixed counterparts, a question of practical importance is when we need to explicitly include these spatial details, and when they can be approximated by a computationally cheaper model. To study this question quantitatively, we first implement a spatially detailed version of the model using the microscale, particle-based simulation software Smoldyn.^20^ We then consider two coarse-grained approximations. The first is the standard well-mixed approximation described in Sec. II C, where we assume that the entire cell is one single, well-mixed reaction volume. The second model is the new multiscale approximation developed in Sec. III, where we use a compartment-based model structure and derive transition rates using first-exit times. For both models, we use diffusion-controlled rates for the bimolecular reactions. In this way, they are directly comparable to the baseline spatial model.

### B. Spatial stochastic simulation using Smoldyn

We first implement the microscale spatial stochastic model using the Smoldyn software.^20^ The underlying model used by Smoldyn is the Smoluchowski diffusion-limited reaction model, in which particles are modeled as hard spheres. Unlike implementations based on Green’s function reaction dynamics,^18,40^ Smoldyn employs approximations and a fixed time step to advance the simulation. Naturally, the accuracy of the simulation depends critically on that time step parameter, ranging from a mesoscopic description for large time steps to increasingly detailed dynamics for small time steps. In our case, for comparison between the modeling levels, it is important that we choose the time step such that Smoldyn’s accuracy is at least at the level of the difference between the well-mixed approximation and the spatial simulation. A very small time step, however, greatly increases the computational cost of the simulation and can become prohibitive in some areas of the parameter space, in particular, where diffusion is very fast. To determine the most suitable time step, we use an adaptive strategy where we successively halve the time step until a satisfactory accuracy is reached. In the ideal case, we would like to stop refining the time step when the gain in accuracy cannot be differentiated from the Monte Carlo error or, in other words, when the Kolmogorov distance between the two consecutive refinements is smaller than the self-distance of the finest refinement, as described in Ref. 41. In practice, this turned out to be too computationally demanding. Instead, we stopped the procedure when the Kolmogorov distance between two consecutive refinements was below some arbitrarily set threshold. Specifically, we compute the Kolmogorov distance over the marginal distributions of each species and report the average of these distances. Thus, the distance between the detailed spatial model and the approximations is not reliable when it is smaller than this threshold. For our study and use case, we found that setting this threshold to 0.1 turned out to be a good compromise between the quality of the results and the computation time.

For completeness, we also estimate the Monte Carlo error by measuring the self-distance of the spatial simulations. These measurements are presented in Appendix B. On an average, the expected self-distance is 0.02, below our threshold of 0.1.

### C. Well-mixed stochastic chemical kinetics

In the well-mixed approximation, diffusion is assumed to be fast enough (relative to reaction binding rates) such that the system has time to become well-mixed between every reaction. The diffusion constant is not an explicit constant in the WMM, but rather enters indirectly via diffusion-limited mesoscopic reaction rate expressions for the bimolecular reaction.

In what follows, we assume that a microscale binding rate, *k*_*a*_, is given for the forward binding reaction (2). The classical Collins–Kimball theory then relates the microscopic rate *k*_*a*_ to the mesoscopic rate $k_{a}^{meso}$,^42^

$$k_{a}^{meso}=\frac{4\pi \sigma Dk_{a}}{4\pi \sigma D+k_{a}},$$ (7)

where *σ* is the sum of the reaction radii of the participating species (another microscopic parameter, used in the Smoldyn simulation). This formula was also more recently motivated by Gillespie,^43^ relating the microscopic reaction parameter to the probability of the reaction given collision. The mesoscopic rate (7) provides a good approximation when the reaction is not too diffusion-limited. An alternative, more accurate mesoscopic rate can be derived by matching properties such as the mean reaction time in the limit of small reaction volumes. In three dimensions, this approach leads to the following diffusion-limited mesoscopic rates:^45,44^

$$k_{a}^{meso}=\frac{k_{a}}{h^{3}}{\left[1+\frac{k_{a}}{D}G\left(h,\sigma \right)\right]}^{-1},$$ (8)

where

$$G\left(h,\sigma \right)=\frac{1}{4\pi \sigma }-\frac{1.5164}{6h}.$$ (9)

In simulations of the WMM and the CBM, we use Eq. (8) as mesoscopic rates for the bimolecular reactions. Since we are considering the system to be well-mixed inside the nucleus, we will take the length scale parameter as $h=V_{n}^{\frac{1}{3}}$ in (8), where *V*_*n*_ is the volume of the nucleus.

### D. Quantitative differences between the spatial and well-mixed models

**FIG. 2.**
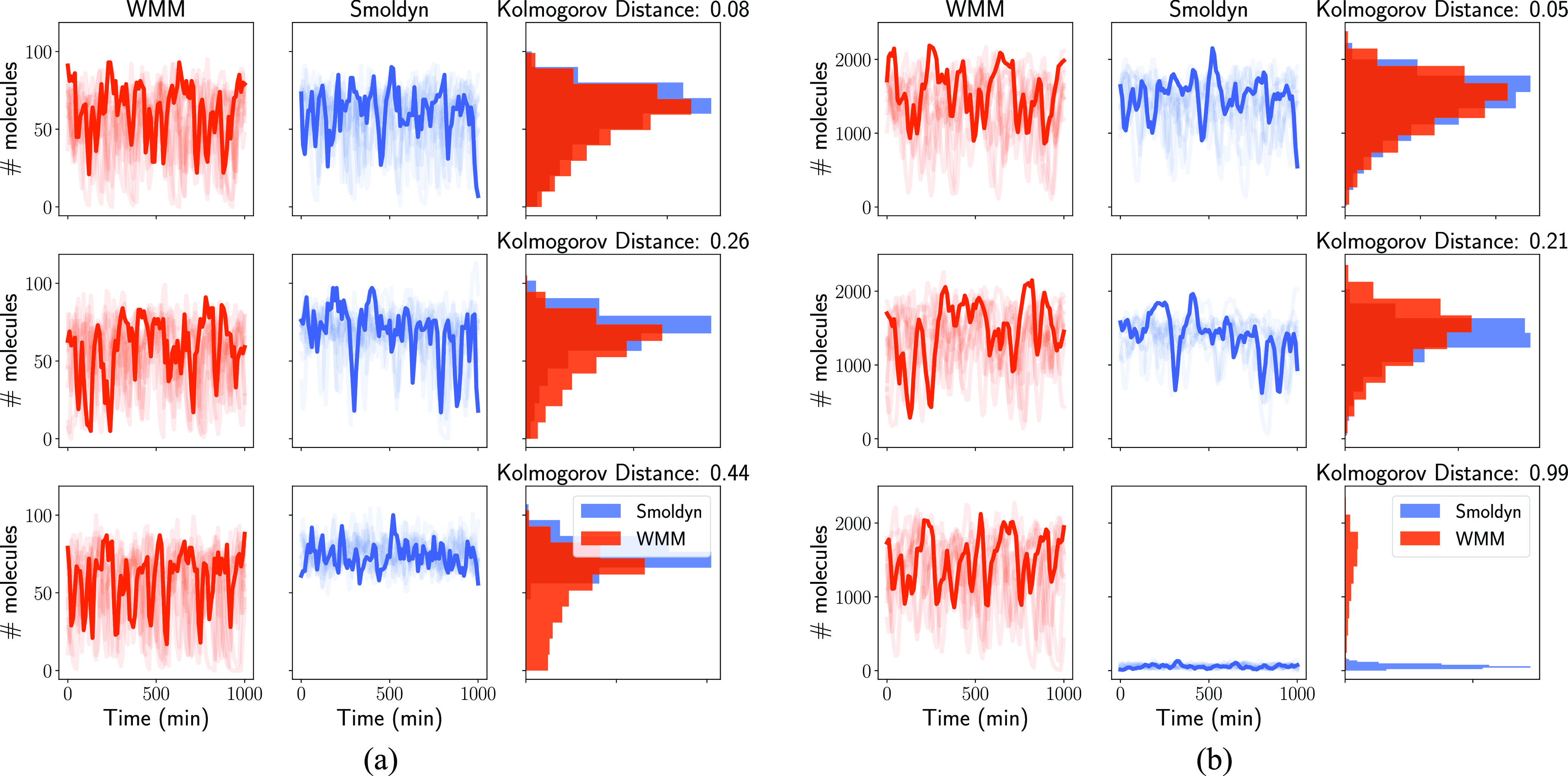
mRNA (a) and protein (b) levels for three different parameter sets: *χ* was set to 1.741 for all simulations, and *D* was set to 3.167, 0.3446, and 0.012 37 *µ*m^2^ min^−1^ for the upper, middle, and bottom rows, respectively. The first two columns show 10 out of 64 simulated trajectories, simulated using either a well-mixed model (first column, red) or Smoldyn (second column, blue). The last column shows the corresponding distribution estimated from these 64 trajectories and the Kolmogorov distance between these two distributions. The total distance is computed by averaging the Kolmogorov distance between the protein distributions and the distance between the mRNA distributions (0.06, 0.24, and 0.72 for the upper, middle, and bottom rows, respectively). Overall, it gives a good sense of when the well-mixed model is a good approximation of Smoldyn.

Figure 2 illustrates the difference between the spatial and well-mixed versions of the model as we vary the ratio between the reaction rates and the diffusion rates (the degree of diffusion control of the system). To preserve the steady state at about the same value in all our simulations, we vary the chemical kinetics parameters together by multiplying them by *χ*, which can be interpreted as the “reactivity” of the system, i.e., the higher the value of this parameter, the quicker the system will change. We use Gillespie’s Stochastic Simulation Algorithm (SSA),^10^ using the implementation provided in the GillesPy2 package^45^ part of the StochSS suite of tools^46^ to simulate the well-mixed model.

To measure the distance between the data distribution of the simulations in Fig. 2, we use the Kolmogorov distance. This metric is commonly used for comparing simulation methods.^41^ Furthermore, Lillacci and Khammash used it as a summary statistic for model selection using flow cytometry data.^47^ It is defined as the maximum difference between the cumulative distribution functions of the two quantities of interest. This distance is also commonly used in statistical tests to determine how likely it is that two datasets are drawn from the same distribution. In our case, each dataset is made of 64 trajectories, each containing 100 samples for each species.

As can be seen in the top row of Fig. 2, the well-mixed approximation works well when the diffusion is fast, i.e., the species’ distributions are well aligned with one another, and the Kolmogorov distance is correspondingly small. In fact, in this case, the Kolmogorov distance is smaller than the refinement threshold we set to determine Smoldyn’s acceptable time step. In other words, it is not possible to say if the difference between the WMM and Smoldyn is due to the well-mixed assumption or the step size choice in Smoldyn. In the second case of medium diffusion (middle row), although mean values are close, there is a clear difference in mRNA and protein distributions, and the Kolmogorov distance increases. In the last case (slow diffusion, bottom row), even mean values are far apart, especially for the case of protein shown in Fig. 2(b), and the Kolmogorov distance is high. This illustrates how the accuracy of the well-mixed approximation depends on the diffusion control of the system and that the Kolmogorov distance captures discrepancies that are not well represented by the mean value, highlighting the stochastic nature of gene expression.

## III. COMPARTMENT-BASED MULTISCALE MODEL

In this section, we introduce a well-mixed compartment-based approximation. Compartment-based models (CBMs) are common enhancements of the simplest well-mixed models. Unlike a spatial model, where the compartments are explicitly represented geometrically, we implicitly account for the two compartments, cytosol and nucleus, by introducing additional reactions in the well-mixed formalism,

$$G_{\emptyset }+P_{nuc}\underset{k_{d}}{\overset{k_{a}^{meso}}{⇋}}G_{P},$$ (10)

$$G_{\emptyset }\overset{\mu }{\to }G_{\emptyset }+mRNA_{nuc},$$ (11)

$$mRNA_{nuc}\overset{k_{\text{exit}}}{\to }mRNA_{cyt},$$ (12)

$$mRNA_{cyt}\overset{\kappa }{\to }mRNA_{cyt}+P_{cyt},$$ (13)

$$P_{cyt}\overset{k_{\text{entry}}}{\to }P_{nuc},$$ (14)

$$mRNA_{nuc}\overset{\gamma_{m}}{\to }\emptyset ,$$ (15)

$$P_{nuc}\overset{\gamma_{p}}{\to }\emptyset ,$$ (16)

$$mRNA_{cyt}\overset{\gamma_{m}}{\to }\emptyset ,$$ (17)

$$P_{cyt}\overset{\gamma_{p}}{\to }\emptyset .$$ (18)

As can be seen, the system can be divided into two logical parts: one for the reactions inside the nucleus and one for those in the cytoplasm. The reactions in each compartment can be simulated in a straightforward way on the well-mixed scale (all the rates can be computed from the corresponding microscopic rates as in Sec. II C). However, the reactions (and their corresponding rates) that model the transport of molecules between the compartments need to be defined. The rate parameters *k*_*exit*_ and *k*_*entry*_ are given by the inverse of the mean first passage time of the corresponding processes. These rates correspond to nucleic mRNA diffusing from the nucleus, through a membrane, and out into the cytosol, and protein diffusing back from the cytoplasm and into the nucleus.

We model this process on the microscopic scale in the following way: An mRNA molecule can diffuse out from the nucleus by reacting instantaneously with the nucleic membrane. A protein *P* can diffuse into the nucleus by reacting, also instantaneously with the membrane from the outside.

We model the nucleus of the cell by a sphere of radius *r*_*n*_, with an absorbing boundary condition. It can be shown that ⟨*T*(*x*_0_)⟩, the expected first-exit time given the initial position of a particle at *x*_0_, is the solution of Eq. (19), where *D* is the diffusion constant. The full derivation of this result is based on Ref. 48 and is described in full length, with its boundary conditions, in Appendix A,

$$D\Delta_{x_{0}}\left\langle T\left(x_{0}\right)\right\rangle +1=0.$$ (19)

Taking advantage of the symmetry of this domain, we solve Eq. (19) to get the expected exit time depending on the initial distance from the sphere’s center *r*. We recover the (well-known) result

$$\left\langle T\left(r\right)\right\rangle =\frac{r_{n}^{2}-r^{2}}{6D},\;\forall r\in \left[0,r_{n}\right].$$ (20)

From this, we obtain the reaction rate

$$k_{exit}=\frac{1}{\left\langle T\left(0\right)\right\rangle }=\frac{6D}{r_{n}^{2}}.$$ (21)

We make the biological assumption that the gene sits roughly in the middle of the nucleus and does not move significantly on the time scale of the simulation. The mRNA is produced at the site of the gene, and for this reason, we assume a fixed point starting location following Refs. 31 and 33. It would also be possible to assume a uniform position in the nucleus, in which case we would take the expected value over the uniform distribution on a sphere of radius *r*_*n*_.

The cytoplasm is modeled by a spherical shell of inner radius *r*_*n*_ and outer radius *r*_*c*_. We set an absorbing boundary on the inner surface and a reflexive boundary on the outside. Again, taking advantage of the spherical symmetry of the domain, we obtain an analytical solution of Eq. (19),

$$\left\langle T\left(r\right)\right\rangle =\frac{r_{n}^{3}r-r_{n}\left(2r_{c}^{3}+r^{3}\right)+2r_{c}^{3}r}{6Dr_{n}r},\;\forall r\in \left[r_{n},r_{c}\right].$$ (22)

We then compute the expected exit time (i.e., the entry time in the nucleus) for a particle whose starting position is selected at random within the domain, that is, the initial position is uniformly distributed across the cytoplasm. This is done by integrating Eq. (22) over the uniform distribution,

$$k_{entry}=\frac{\int_{\Omega }\text{d}x_{0}}{\int_{\Omega }\left\langle T\left(x_{0}\right)\right\rangle \text{d}x_{0}}=\frac{15r_{n}\left(r_{c}^{3}-r_{n}^{3}\right)D}{5r_{n}^{3}r_{c}^{3}-9r_{n}r_{c}^{5}+5r_{c}^{6}-r_{n}^{6}}.$$ (23)

An mRNA molecule immediately starts producing P molecules after exiting the nucleus. This implies that on the microscopic scale, P molecules will be produced close to the membrane for some time, until the mRNA has had time to become well-mixed inside the cytosol. In turn, this also implies that the P molecules will, possibly, diffuse back into the nucleus quickly.

Since we assume molecules to always be well-mixed inside their domains in the compartment-based model, we cannot simulate this process with high accuracy if the production of P molecules in the cytosol and the entry rate are too fast. Intuitively, we should have a mesoscopic entry rate that is slower than the time that it takes for the molecule to get well-mixed, which is proportional to $V_{cyt}^{2/3}/6D$. In Sec. IV, we investigate the parameter domain where the well-mixed model (WMM) and the new compartment-based model (CBM) accurately approximate the microscopic particle simulation.

## IV. NUMERICAL EXPERIMENTS

In this section, we assess the accuracy and limitations of our proposed multiscale approximation strategy and study the potential performance gains. First, we look at the errors in a chosen set of summary statistics and by comparing distributions via the Kolmogorov distance. Then, we demonstrate a practical application of the approximation in a likelihood-free parameter inference setting.

### A. Approximation quality

We generate 256 parameter points from a 16 × 16 grid spanning our 2D parameter space. For each parameter point, we simulate each model level (well-mixed, compartment-based, and fully spatial) and compare the results using the Kolmogorov distance metrics described in Subsection II D and by comparing the distance between a set of summary statistics.

Summary statistics such as moments are commonly used in likelihood-free inference to reduce data dimensionality and are critical to ensure good efficiency in the context of Bayesian parameter inference.^49^ Choosing good or even optimal summary statistics is a hard problem in practice. For the sake of simplicity, we here use a set of four commonly used summary statistics: the mean value, the standard deviation, the minimum value, and the maximum value. We apply these statistics to the two species of interest in our model (P and mRNA, using the total amount in the entire cell for both species) and normalize these values, before using the *L*_2_ norm to compute the final distance between two simulations.

Each simulation consists of an ensemble of 64 statistically independent realizations. The simulation process can be described as follows: we first generate the initial state by running the simulation for 1000 min using the base parameters from Table I, in order for the simulation to reach the steady state. We run the simulation for 1200 min using the current parameters and discard the first 200 min as burn-in. Each of the 64 trajectories then contains 100 time samples. We then extract the marginal distributions of both mRNA and proteins and use these 2 × 6400 values to compare each model.

We ran all simulations on Rackham, a High Performance Computing (HPC) cluster provided by the Multidisciplinary Center for Advanced Computational Science (UPPMAX). Each node on Rackham is equipped with two 10-core Intel Xeon E5 2630 v4 at 2.20 GHz/core and 8 × 16 384 MB (128 GB) of ECC 2400 MHz DIMM DRAM memory. Each simulation was run on a single core. Simulation time ranged from 2.5 to 23.1 s for the WMM, from 2.5 to 34.0 s for the CBM, and from 240.8 s to 34 h 28 min for Smoldyn, depending on parameter values. In all cases, the shortest runtimes were obtained when both the diffusion and the reaction rates were minimal, while the longest runtimes were reached when these parameters were maximal.

**FIG. 3.**
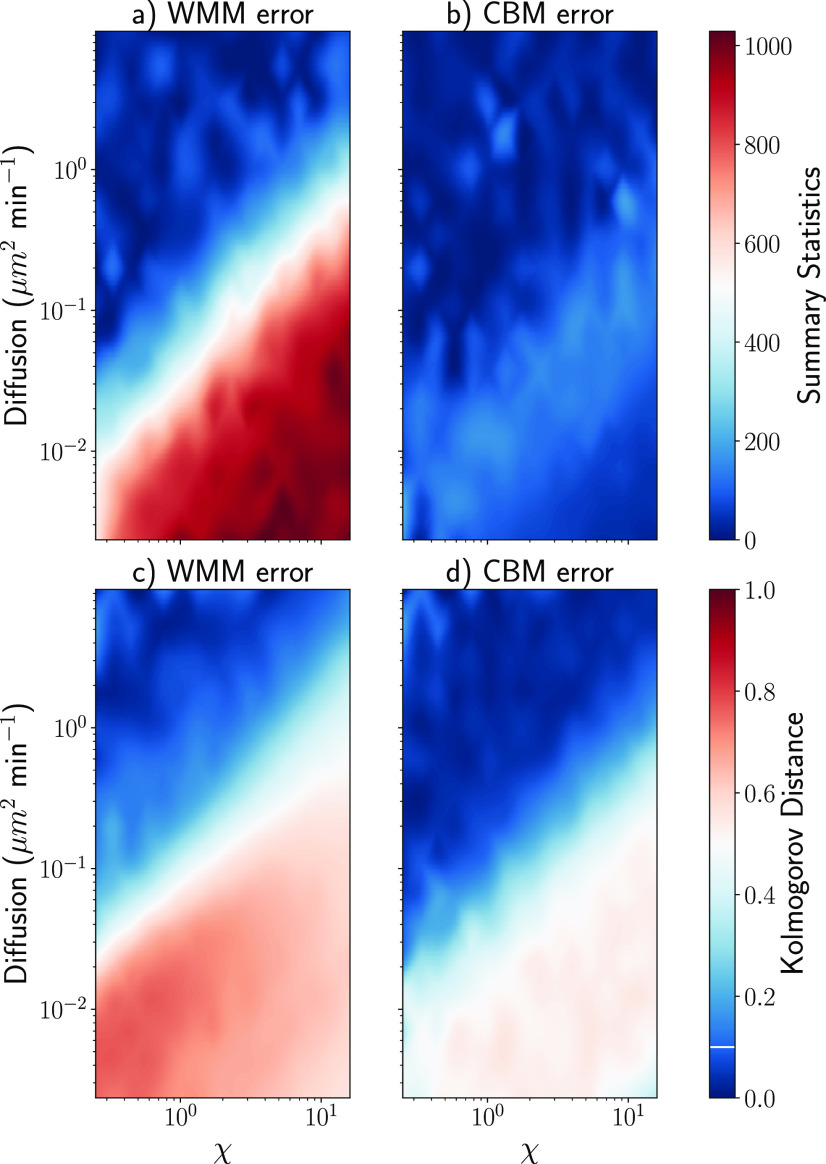
Measured error between Smoldyn and the WMM [(a) and (c)] and the CBM [(b) and (d)], using either summary statistics [(a) and (b)] or the Kolmogorov distance [(c) and (d)], depending on parameters *D* and *χ*. In all four cases, accuracy decreases as diffusion decreases and *χ* increases. Overall, the CBM is significantly more accurate than the WMM, especially when using summary statistics, when diffusion is low and *χ* is high. The white line on the color bar corresponds to the threshold for selecting Smoldyn’s step size. Every difference below this threshold is not significant.

As expected, the well-mixed approximation is accurate down to Smoldyn’s step size error compared to the spatial model provided the diffusion is high enough compared to the reactivity of the system. Conversely, as diffusion slows down, the well-mixed approximation loses accuracy. Figures 3(a) and 3(c) provide quantitative insight into the validity of the well-mixed approximation for this model system. While the simple well-mixed model attempts to incorporate effects from diffusion via the diffusion-controlled rate (8), it is inherently limited by the failure to resolve the spatial details of the model (e.g., the relative size and shape of the nucleus and the cytoplasm).

The CBM also relies on the well-mixed approximation (although for smaller domains, where this approximation is more likely to hold true), and we expect a similar trend in accuracy to the WMM but with an improved range of validity. Figures 3(b) and 3(d) show the summary statistics distance and the Kolmogorov distance between Smoldyn and the CBM. Similar to the well-mixed model, the approximation deteriorates as diffusion slows down and reaction rates increase, although this happens in a smaller section of the parameter space compared to the WMM. In addition, in the case of summary statistics, this trend is much less visible than with the Kolmogorov distance. In other words, it seems that the compartment-based model is accurate up to the second order moment for the cross section of the parameter space we have studied.

We can divide the cross section of the parameter space into three main regions: in the upper left corner, the well-mixed approximation is good enough and both the CBM and the WMM show results close to the particle simulation. In the bottom right corner, where diffusion is very slow and reactions are fast, neither the CBM nor the WMM approximates Smoldyn well; the error is notably higher although there is a significant improvement in the CBM compared to the WMM. In between these two regions lies a transition area, where the approximation is good enough with the CBM but not with the WMM. This range is large in the case of the summary statistics we consider. In summary, the CBM extends the region with acceptable accuracy comparable to Smoldyn substantially compared to the WMM.

These results also provide quantitative information regarding which model to use depending on the parameter of the system: roughly speaking, based on these experiments, if the ratio between the diffusion and the transcription rate *D*/*χ* is below 0.05, we can expect Smoldyn to be the best option. If this ratio is between 0.05 and 0.1, the CBM becomes the best choice (based on its significantly faster runtime). Finally, if the ratio is above 0.1, all models show comparable accuracy.

**FIG. 4.**
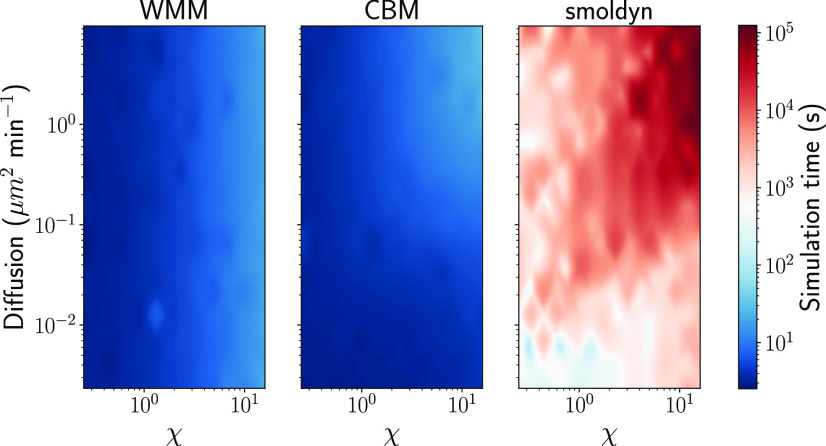
Simulation time for all three models, depending on parameters *D* and *χ*. Overall, when the WMM or the CBM matches Smoldyn’s accuracy, it can be expected to be two or three orders of magnitude faster.

The gain in accuracy of the CBM over the WMM would not be of any practical use if it translated to a significant increase in computational cost. In Fig. 4, we show the computation times of all three models across the same cross section of parameter space. For Smoldyn, we only record the time necessary to run the simulations with the finest, acceptable time step, i.e., we discard the time spent adapting the simulation to find this time step. We recall that the time steps are chosen such that the self-error between two successive time step halvings is below the 0.10 threshold. Our results show that both the WMM and the CBM remain two or three orders of magnitude faster than Smoldyn across the whole parameter subspace studied in this paper.

### B. Likelihood-free parameter inference using approximate Bayesian computation

Next, we consider the task of inferring the reaction rate parameters in the Gene Regulatory Network (GRN) model from time series data. Specifically, we generate data using the fine-scale spatial model and then study how parameters inferred using the WMM compare to those obtained using the CBM.

Approximate Bayesian Computation-Sequential Monte Carlo (ABC-SMC) is a popular likelihood-free parameter inference technique commonly used in systems biology. The method relies on systematic comparisons of the observed data and the data generated by simulating the system with parameters drawn from *a prior* distribution. Parameter sets that lead to a small distance (as determined by a threshold parameter) between simulated and observed data are retained and are used to form the *posterior* distribution.^50^ The high-dimensional nature of the time series data leads to a high variance, so in practice, the distance is usually computed based on summary statistics, such as the statistics demonstrated in Fig. 3, or the Kolmogorov distance.^47^

The choice of the distance threshold is also important for both accuracy and performance; if it is too large, many parameter sets are retained and the posterior does not improve much from the prior, and if it is too small, the computational cost of simulations becomes prohibitive due to low acceptance rates, especially if the simulator is expensive. In ABC-SMC, successive sets of samples are generated with finer and finer thresholds to approximate the true posterior distribution of the parameters to be estimated.^51^ To compute each set (called a population), a new parameter point (called a particle) is sampled from the distribution of the previous population (now used as the prior distribution). Once enough particles have been accepted in the current population, the threshold is refined and a new population is generated. The final result is taken as the posterior distribution. ABC-SMC can also be used to compare several alternative models. In this case, the model used to simulate the data is represented by an extra parameter to be inferred. The posterior distribution of this parameter then gives the probability of each model, given the data.

Here, we conduct such a model comparison to highlight how the CBM can be used as a cheap surrogate of a full spatial simulation, capturing data better than the well-mixed model. For this experiment, we use the Python framework pyABC.^52^ Synthetic observed data are generated with Smoldyn and comprise 64 trajectories, each with 100 time samples. We use the summary statistics from Sec. IV A and the Euclidean distance to measure the difference. All simulation parameters are kept the same as those in the previous experiment. We let pyABC generate ten populations, starting from a uniform prior and using the default strategy to refine the thresholds (namely, MedianEpsilon, which sets the next threshold to the median of the distances of the current population).

**FIG. 5.**
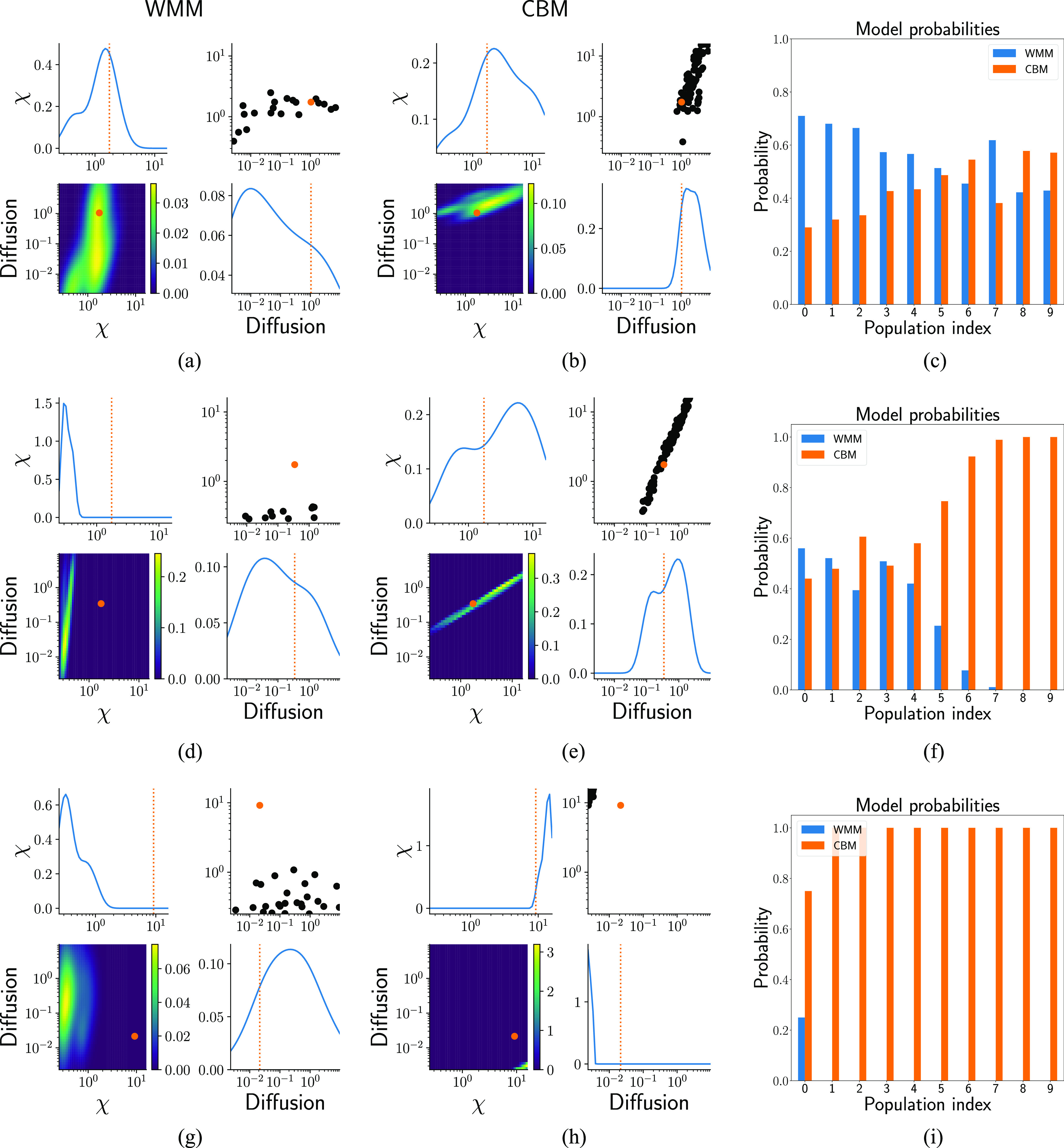
Posterior distributions computed using ABC-SMC as implemented in pyABC, using synthetic data generated with Smoldyn for three different possible parameter points. Summary statistics were used to measure the distance between the candidate particles and the synthetic data. The posteriors were computed using either the WMM [left column; (a), (d), and (g)] or the CBM [middle column; (b), (e), and (h)]. The orange dots and vertical lines denote the true parameter values used to generate the observed data. The right column [(c), (f), and (i)] shows the posterior probabilities assigned to each model. For the first parameter set, both models result in relatively good estimates of the true parameters [(a) and (b)], and we are not able to select a clear preferred model (c). For the second parameter set (middle row), the CBM posterior results in relatively accurate inference, while the WMM gives poor results. The CBM is selected as the clearly favored model (f). For the third parameter set (last row), both models struggle to recover an accurate posterior mean, although the CBM is still heavily favored in terms of model probabilities, highlighting the fact that it captures critical features of the spatial dynamics.

Figure 5 summarizes the results. Each row shows the posterior estimate for a different synthetic dataset. The true parameters used to generate the observed data are shown with orange dots and vertical dotted lines. The first and second columns show the posterior distributions generated from the last population with the WMM [(a), (d), and (g)] and the CBM [(b), (e), and (h)], respectively. In the case where the last population did not contain enough particles, the last population containing more than ten particles was used. The last column [(c), (f), and (i)] shows the model probabilities for each model.

For the first parameter set (first row) when the diffusion is fast (reaction-limited reactions), both the WMM and the CBM provide acceptable estimates of the true parameters and neither model is clearly favored. In this regime, the standard WMM model is an acceptable approximation to the spatial dynamics from the perspective of inferring parameters. For the second parameter set (second row), the system becomes diffusion-limited and the spatial effects become more prominent. As can be seen, the CBM provides a reasonable estimate of the true parameters, while the WMM yields poor results. By the time the last population is complete, the CBM is heavily preferred by pyABC (f). For the final parameter point in the strongly diffusion-limited regime (third row), neither model results in a good estimate, although the CBM is still strongly preferred over the WMM (i).

Clearly, given a sufficient computational budget, using the Smoldyn simulator during inference, should result in even better parameter estimates, at the cost of much longer computational times. To estimate this cost, we sampled the computational time of the three solvers across the studied parameter space in Fig. 4. As can be seen, the detailed spatial model requires one to several orders of magnitude longer simulation time depending on parameters. We then looked at the accepted particles from the three experiments presented in Fig. 5 and interpolated their cost for each of the three solvers, in terms of computation time, using the data from Fig. 4. The results are presented in Table II. Since the sampling particles for each population are an embarrassingly parallel problem, we express the cost estimate in terms of core hours. However, note that this is the estimated cost of the accepted particles only; hence, it is a lower bound of the true cost. Indeed, it does not include the overhead associated with the pyABC software, the non-parallelizable sections of the ABC-SMC algorithm, or the time associated with rejected particles (which are not included in the pyABC output). For our experiments underlying Fig. 5—where we used a mixture of simulations from the WMM and the CBM—the total cost including accepted and rejected particles ranged from 10 to 100 core hours. It is clear that the estimated minimal cost of an inference experiment with either the WMM or the CBM is several orders of magnitude lower than the cost associated with Smoldyn. At the same time, the cost of using the CBM only is marginally higher that that using the WMM, highlighting the substantial potential gains from using a surrogate model.

**TABLE II.** Estimated minimal execution cost for an ABC-SMC experiment using either of the three solvers, for three pairs of values (*D*, *χ*): high diffusion (1.04, 1.74), medium diffusion (0.32, 1.74), and low diffusion (0.02, 9.19). For practical reasons, the time is estimated from the accepted particles collected by pyABC and thus provides a lower, ideal bound to the actual time it would take to run these experiments.

	Estimated minimal cost (core hours)		
Solver	High diffusion	Medium diffusion	Low diffusion
WMM	1.70	1.47	3.49
CBM	2.21	1.79	1.14
Smoldyn	4635.48	3405.71	780.40

Overall, our results show that using the compartment-based approach does increase the accuracy of the well-mixed approximation, both generally in terms of the error for selected summary statistics and marginal distributions (Fig. 3) and in the context of parameter inference tasks using state-of-the-art likelihood-free methods (Fig. 5) As expected, the errors in the approximation depend on the degree of the diffusion control of the system, which we highlight by varying parameters in the space of the diffusion rate and the reactivity of the system. Importantly, we show that the CBM, compared to the WMM, provides a boost in accuracy for diffusion-controlled systems resulting in much more accurate parameter inference in this regime, at a marginal increase in computational cost.

## V. DISCUSSION

The well-mixed assumption is a common approximation in computational systems biology, and its theoretical framework is well established. However, spatial dynamics can have a strong influence on the dynamics. In practice, it is important for large-scale modeling tasks such as model exploration and parameter inference to use the cheapest possible simulation method that can accurately capture the behavior of interest. We compare the classic well-mixed approximation and a fine-grained spatial particle-based simulation for a negative-feedback regulatory network and propose a new computationally cheap compartment-based multiscale approximation that greatly extends the well-mixed model’s domain of applicability. We focused on the two parameters that influence the degree of well-mixedness of the system, namely, the diffusion rate and the speed of the chemical reactions. Our results reveal in which area of the parameter space the well-mixed assumption can be relied on, and they show a clear advantage for the compartment-based model.

We obtain an *a priori* parametrization of the proposed multiscale model by relying on a concentric sphere geometry for the cell. However, we note that in the case of a general geometry, the reaction rates in the compartment model could be readily obtained by solving Eq. (19) numerically. A clear advantage of the concentric sphere geometry used in this study is that we are able to derive analytical formulas for the transition rates between compartments. This makes it possible to cheaply parametrize the model for any parameter combination. This geometry is an important case; it is by far the most commonly considered geometry in spatial models of eucaryotes, and it is also used in the overlapping-sphere model^53^ for multicellular simulation. A recent study used such an overlapping-sphere model together with particle simulations to study the effect of spatial dynamics in gene regulation on receptor activation and its role in tumor growth.^32^ A future envisaged application area of the multiscale compartment model developed herein is a means to add spatial stochastic detail in such simulations with only a moderate increase in the computational cost.

We here considered two compartments—one for the nucleus and one for the cytoplasm. It is of course possible to add more compartments, either to model other spatial features of the cell or to refine the position of each particle in the cell, in a similar fashion to the RDME framework.^14^ In this case, the transition rate from one compartment to another would be computed by first setting a Dirichlet boundary condition at the interface between these compartments and a Neumann boundary condition everywhere else, and then solving Eq. (19). Thus, the same domain with different boundary conditions would be used to get the transition rates from one compartment to all its neighbors. We note, however, that the objective here is to use very few compartments to obtain a cheap approximation, rather than to provide an alternative to a resolved RDME simulation, which would also become prohibitively expensive in the type of parameter inference task we demonstrate here.

We chose a model of negative-feedback gene regulation as a model problem in this study. While here cast in generic terms, we would like to emphasize that by including the spatial dimension, the size of the model is quite representative for models previously considered in applied spatial modeling projects,^4,30^ and in particular, it is identical to a model of the Hes1 dynamics^31^ (but there parameterized to compare to a specific dataset). Although subject to future studies, we believe that the method will generalize to larger, more complex networks. For example, Mitogen Activated Protein Kinase (MAPK) pathways are structurally very similar to the model we use here when it comes to the spatial aspects. The key difference would be additional species and more reactions in the cytoplasm (the phosphorylation cycles). However, the actual dynamics regarding the transport to the nucleus could be modeled in almost the same way as our example, and this is an important reason for why we chose this model problem. Our approach is best suited for the scenario where the key spatial aspect is the diffusion-limited transport between main cellular compartments such as the cytosol to the nucleus as it captures the mean first passage times accurately. It is less suited for scenarios where fine-grained localized spatial correlations between molecules have large direct effects; i.e., for highly diffusion-limited scenarios a fully resolved spatial model will be needed, as is expected and can be seen in our numerical results.

We chose to compare models using two metrics, the first based on a small set of summary statistics and the second on the Kolmogorov distance. Summary statistics are a common tool in the Bayesian likelihood-free inference. Using a different set of summary statistics would influence the measured error between our models. For instance, we expect the possibility to differentiate between the different modeling levels in the model selection we conduct to depending on both the summary statistics and the amount of data available. This is itself an interesting question and will be investigated in a future study. We also note that compared to only considering the mean values, the Kolmogorov distance better captures the dynamics involved in our system (see Fig. 2). Kolmogorv distance has also been used for Bayesian inference and model selection from fluorescent activated cell sorting (FACS) data in Ref. 47 as a metric to compare simulated data to experimental data. For the sake of simplicity, we only used synthetic data for this study, but a similar approach with experimental data would be relatively straightforward following the same protocol.

Finally, we want to comment on the proposed CBM approximation vs RDME simulations, which would fall in between Smoldyn and the well-mixed models in cost, provided the mesh resolution is not too fine. It is natural to think that a very coarsely resolved RDME simulation would compare favorably to this approximation. However, this is not likely for two main reasons: (1) The overhead of handling meshes and the many diffusion jumps for larger diffusion constants become relatively large compared to a highly optimized well-mixed code. (2) Our approximation relies on analytical hitting times accounting for the actual geometry, and these can be expected to perform better than a RDME approximation using very large mesh elements (which would not be able to resolve the boundaries in a reasonable way). Even in the case that a coarse RDME simulation would have about the same accuracy as the CBM appromixation, it would be slower and much more complex to setup, so it would be outside its “sweet spot” in terms of efficiency/fidelity trade-off (medium to high spatial resolution). Furthermore, here we are seeking approximations that have a very small software complexity footprint (to facilitate, e.g., future integration in muticellular simulations). RDME software for non-cubic domains intrinsically has a need for mesh generation and assembly of diffusion jump coefficients. We want to avoid this in this work, to arrive at a very cheap model that carries the key spatial features without all the engineering complexity. A distinct advantage with the proposed approximation is that it can be readily simulated with any well-mixed code. This means that it can readily be used also in inference packages such as Sciope^54^ that supports Gillespy2.^45^

## Figures and Tables

**FIG. 6.**
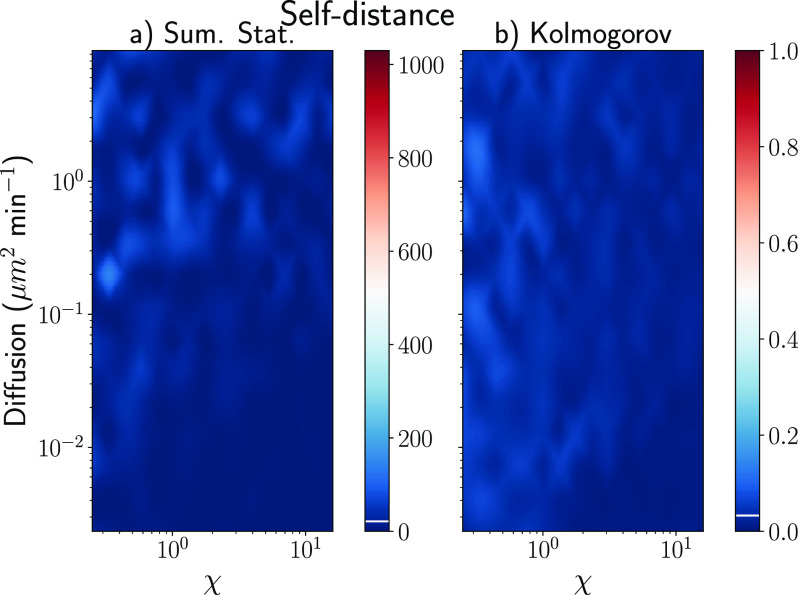
Self-distance between two sets of 32 trajectories and 100 time samples, using both the Euclidian distance based on summary statistics (a) and the Kolmogorov distance (b). The white line shows the average self-distance (21.2 for the summary statistics and 0.033 for the Kolmogorov distance). With 64 trajectories, the average distances can be expected to be around 15.0 with summary statistics and 0.023 with the Kolmogorov distance. In the case of the Kolmogorov distance, this is much below the threshold we used to select Smoldyn’s step size (0.1).

## Data Availability

Data sharing is not applicable to this article as no new data were created or analyzed in this study.

## APPENDIX A: HITTING-TIME DERIVATION

### 1. Equation for the first-exit time

In order to derive closed-formed formulas for the exit and entry rates, let us consider a particle starting at position *x*_0_ and diffusing in some domain Ω, with an absorbing boundary Γ_*a*_ and a reflexive boundary Γ_*r*_, and let *X*(*t*) be the position of the particle at time *t*. Additionally, let us denote *P*_*X*_(*x*, *t*|*x*_0_, *t*_0_), the probability distribution of *X*, and *T*(*x*_0_), the random time when the particle hits the boundary of the domain.

Let us now consider *G*(*x*_0_, *t*), the probability that a particle starting at *x*_0_ is still in Ω at time *t*,

$$G\left(x_{0},t\right)=\int_{\Omega }P_{X}\left(x,t|x_{0},0\right)dx.$$ (A1)

Put differently, 1 − *G*(*x*_0_, *t*) is the probability that the particle has already exited the domain at time *t*, i.e., *P*(*T*(*x*_0_) ≤ *t*). By definition, this is the cumulative distribution function of *T*(*x*_0_); hence, the probability distribution function of *T*(*x*_0_) is given by

$$P_{T}\left(t;x_{0}\right)=-\frac{\partial G\left(x_{0},t\right)}{\partial t}.$$ (A2)

The expected first-exit time is then given by

$$\left\langle T\left(x_{0}\right)\right\rangle =\int_{0}^{\infty }tP_{T}\left(t;x_{0}\right)dt=\int_{0}^{\infty }t\left(-\frac{\partial G\left(x_{0},t\right)}{\partial t}\right)dt.$$ (A3)

Integrating by parts, it can be shown that the following equation holds:

$$\left\langle T\left(x_{0}\right)\right\rangle =\int_{0}^{\infty }G\left(x_{0},t\right)dt.$$ (A4)

Following Ref. 48, we now write down Einstein’s Fokker–Planck equation, also known as the Smoluchowski equation,

$$\frac{\partial }{\partial t}P_{X}\left(x,t|x_{0},0\right)=D\Delta_{x}P_{X}\left(x,t|x_{0},0\right),$$ (A5)

subject to the initial condition

$$P_{X}\left(x,0|x_{0},0\right)=\delta \left(x-x_{0}\right),\;x_{0}\in \Omega$$ (A6)

and the following boundary conditions:

$$P_{X}\left(x,t|x_{0},0\right)=0,\;\forall t\geq 0\;\forall x\in \Gamma_{a}\;\forall x_{0}\in \Omega ,$$ (A7)

$$\frac{\partial }{\partial x}P_{X}\left(x,t|x_{0},0\right)=0,\;\forall t\geq 0\;\forall x\in \Gamma_{r}\;\forall x_{0}\in \Omega ,$$ (A8)

where Δ_*x*_ is the Laplacian operator according to variable *x*.

Our system being temporally homogeneous (i.e., the dynamics of the system do not depend on some external clock), we can write the equivalent backward Fokker–Planck equation, where the Laplacian is now computed with respect to *x*_0_,

$$\frac{\partial }{\partial t}P_{X}\left(x,t|x_{0},0\right)=D\Delta_{x_{0}}P_{X}\left(x,t|x_{0},0\right).$$ (A9)

With boundary conditions,

$$P_{X}\left(x,t|x_{0},0\right)=0,\;\forall t\geq 0\;\forall x\in \Omega \;\forall x_{0}\in \Gamma_{a},$$ (A10)

$$\frac{\partial }{\partial n}P_{X}\left(x,t|x_{0},0\right)=0,\;\forall t\geq 0\;\forall x\in \Omega \;\forall x_{0}\in \Gamma_{r}.$$ (A11)

We can now integrate this equation over Ω, which gives

$$\frac{\partial }{\partial t}G\left(x_{0},t\right)=D\Delta_{x_{0}}G\left(x_{0},t\right).$$ (A12)

We finally integrate this equation in time,

$$\int_{0}^{\infty }\frac{\partial }{\partial t}G\left(x_{0},t\right)dt=D\Delta_{x_{0}}\int_{0}^{\infty }G\left(x_{0},t\right)dt,$$ (A13)

which gives us

$$D\Delta_{x_{0}}\left\langle T\left(x_{0}\right)\right\rangle +1=0.$$ (A14)

### 2. Boundary conditions

Putting (A1) and (A4) together, note that

$$\left\langle T\left(x_{0}\right)\right\rangle =\int_{0}^{\infty }\int_{\Omega }P_{X}\left(x,t|x_{0},0\right)dxdt.$$ (A15)

From (A10), we get

$$\left\langle T\left(x_{0}\right)\right\rangle =0,\;\forall x_{0}\in \Gamma_{a}.$$ (A16)

From (A11), we get

$$\frac{\partial }{\partial n}\left\langle T\left(x_{0}\right)\right\rangle =0,\;\forall x_{0}\in \Gamma_{r}.$$ (A17)

## APPENDIX B: SELF-DISTANCE

Figure 6 shows self-distance between two sets of 32 trajectories and 100 time samples, using both the Euclidian distance based on summary statistics and the Kolmogorov distance.
